# Tracheotomy in a Case of Croup Successful

**Published:** 1853-07

**Authors:** William Craig


### SURGERY.

*Tracheotomy in a Case of Croup Successful.* By William Craig, Esq.—The subject of the following case was Master William A., aged 7. He is of a delicate and somewhat irritable constitution, and has had enlarged tonsils since he was three years of age. The enlargement of the tonsils has not been uniform, but liable to variations in size, from occasional exposures to cold. The frequent application of a weak solution of nit. argent. had only a partially beneficial effect, as the tonsils always continued less or more swollen.

To invigorate his constitution, and improve the affection of the throat, he was brought from Glasgow to the coast, to obtain the benefit of the sea air. I saw the little patient for the first time on the 23d of March. I then found the tonsils more than usually red, and pretty much swollen, and, on the most projecting part, they were partially ulcerated. I touched the ulcerated portion of the tonsils lightly with nit. argent., and recommended counter-irritation around the neck.

On entering his room on the following day, the first sound that met my ear was the well-known ringing cough of croup. My little patient was immediately subjected to the most rigid antiphlogistic treatment. Emetics and purgatives were freely administered, warm salt placed round the throat, and leeches applied during the first day of these croupy symptoms. On the second day, no improvements being apparent, the emetics were given in more decided doses; but their effects were more depressing than emetic. Besides antimony, ipecacuanha and sulphate of zinc were perseveringly administered by anxious and trustworthy attendants. Calomel was also given in repeated doses, accompanied by mercurial frictions, but without any specific or beneficial effect. A blister was also applied to the side of the neck. Notwithstanding all these measures, the disease progressed to the full developed stage. On the fourth day of the disease, the emetics and other measures were continued, without the slightest melioration of the symptoms, as every repeated paroxysm returned with aggravated violence, and threatened immediate suffocation. I had had a dread from the first that the case might come to this extremity, and had resolved—and had the concurrence of Dr. Paterson, from Glasgow, in the propriety of my resolution—to give my patient the benefit of tracheotomy as a *dernier resort.* Dr. Whiteside, of this town, was also of opinion that my patient should have the chance of the operation; and he assisted me in the performance of it. The boy was seated on the knee of an assistant. A free incision of fully two inches in length was then made, without losing a drachm of blood; and every drop was removed before opening the trachea. It was opened to the extent of an inch fully, and the incision was at the same time carried through the false membrane. Immediately on the opening being made, the membrane was seen vibrating in the trachea, and a violent expiratory effort caused a large portion of it to be forced out; and the patient was immediately and completely relieved. The larger portion of the membrane thus ejected was about two inches in length and, at its greatest breadth, was fully an inch and a quarter. There were two small fragments thrown out at the same time, which, when added to the largest piece, made it of a uniform length and width. It was fully a line in thickness, and, in tenacity, it somewhat resembled that of an orange skin.

For a number of hours after the operation, there was little cough and no expectoration; but there was a considerable discharge for some time after this, without any bronchial or other pulmonary irritation which could be discovered to account for it. The discharge might, in my opinion, be supplied for the most part by the solution of that portion of the membrane which lined the trachea between the glottis and the opening. On the third day after the operation, the tube was found more than usually obstructed; and, on removing it, a portion of thin, membranous matter was flapping in the wound. Part of this was forced out by the expiratory efforts; and another portion adhered so firmly, that some force was necessary to separate it from the attachment it had with the inner surface of the trachea above the wound. This was evidently that portion of the false membrane which filled the trachea between the glottis and the upper part of the wound. A portion of it had a distinct tubular form; but it was remarkably attenuated, having rapidly dissolved after the opening through the trachea had been effected. In the course of eight days after the operation, the natural air passage had become so clear, that the tube was removed, and the respiration was established in its natural course. There was a partial return of the croupy cough on the 22d of April; but it became speedily better, and he has since continued to progress most satisfactorily, and is now nearly well, and the wound in the neck is now cicatrised. There was some suspicion that this attack was connected with scarlatina, as a younger sister of our little patient died at the same house from a severe attack of fever, accompanied by the species of sore throat peculiar to this affection. What, in addition, gave countenance to this apprehension, was the desquamation, to some extent, of the cuticle, from the anterior part of the chest; but this might have depended exclusively on an erysipelatous condition of the skin around the incision, and extending down the anterior part of the chest a few days after the operation. The age of the patient, and the uncomplicated condition of the complaint—there was no pulmonary affection—were very encouraging circumstances in this case. Another circumstance which, in my opinion, promoted a favorable result, was the free opening in the trachea, as a strong expiratory current was permitted, which forced out the whole of the false membrane which was situated below the incision. The false membrane reached about an inch below the lowest point of the incision, very near the bronchial bifurcation. Some authors mention, that there is no prospect of success if the membrane extend below the point chosen for incision.

I do not consider that the question of tracheotomy should be delayed till the leaden hue of the countenance and purpled color of the lips evince to what extent the vitiation of the circulating fluid has advanced. The hopelessness of the case can be easily prognosed before the affection has advanced so far; and if an earlier period were generally chosen, there would, in all probability, be less chance of the extension of the false membrane into the bronchial tubes. It would have been hopeless to have expected the expulsion of the false membrane through the glottis, when, even in the attenuated condition in which the last portion came away, it required considerable traction with forceps to separate, or, rather, tear it from its adhesion to the inner surface of the trachea. Though the membrane had been loose in the trachea, such a mass coming up through the glottis with diminished expiratory efforts to force it through would inevitably have produced suffocation. Although the lower edge of the membrane was unattached to the walls of the trachea, it was firmly connected with the upper part, as was evinced by the strong adhesion of the attenuated portion that came last away.

The greatest number of authors who write on this disease consider tracheotomy as a hopeless expedient to save patients laboring under this formidable malady. If one can be saved out of ten, or even a much smaller exceptional proportion, I see no good grounds to withhold from a little sufferer the only chance which he has of being snatched from the jaws of death.—*Med. Times and Gaz.*

